# Heterogeneous Pathology of Melasma and Its Clinical Implications

**DOI:** 10.3390/ijms17060824

**Published:** 2016-05-26

**Authors:** Soon-Hyo Kwon, Young-Ji Hwang, Soo-Keun Lee, Kyoung-Chan Park

**Affiliations:** 1 College of Medicine, Seoul National University, Jongro-gu, Seoul 03080, Korea; 2 Seoul National University Bundang Hospital, Seongnam-si, Gyeonggi-do 13620, Korea; soonhyo17@hanmail.net (S.-H.K.); 1polly@hanmail.net (Y.-J.H.); drleesookeun@gmail.com (S.-K.L.)

**Keywords:** basement membrane, histopathology, mast cells, melasma, photoaging, vascularization

## Abstract

Melasma is a commonly acquired hypermelanosis that affects sun-exposed areas of the skin, with frequent facial involvement. Its histologic manifestations are evident in the epidermis, extracellular matrix, and dermis. In addition to epidermal pigmentation, pathologic findings of melasma include extracellular matrix abnormality, especially solar elastosis. The disrupted basement membrane has been described in melasma with variable incidences. In the dermis, an increase in vascularity and an increase in the number of mast cells were observed, indicating that dermal factors have critical roles in the pathogenesis of melasma, despite the fact that melasma is characterized by epidermal hyperpigmentation. This review discusses such histologic characteristics of melasma, with consideration to their implications for melasma treatment.

## 1. Introduction

Melasma is an acquired hypermelanotic condition presenting with light-to-dark brown-colored irregular macules on sun-exposed areas of the skin, especially that of the face [1]. It is particularly common in women in their thirties and forties, especially in Asians [2]. Chronic ultraviolet (UV) exposure, genetic factors, and sex hormones are generally believed to be involved in the occurrence of melasma [3,4,5,6]. However, the pathogenesis of melasma has not yet been fully elucidated.

The management of melasma is usually challenging. It is often recalcitrant to treatment and often recurs despite successful treatment [7]. To better understand such challenges, including its likelihood of recurrence, histologic variation of melasma is herein discussed. Histopathologic features of melasma reported in the literature thus far, albeit limited in scope, may provide insights to better comprehend treatment responses. This review discusses diverse histologic features of melasma, such as solar elastosis, basement membrane disruption, increased vascularization, and an increased number of mast cells, and their possible implications with respect to melasma treatment.

## 2. Heterogeneous Histologic Findings of Melasma

### 2.1. Dermal Extracellular Matrix (ECM) Abnormality (Solar Elastosis)

Although melasma is characterized by epidermal pigmentation, dermal extracellular matrix (ECM) abnormalities are commonly observed. Solar elastosis, an accumulation of abnormal elastic tissues in the dermis due to prolonged sun exposure—a process known as photoaging—has been a frequently described feature in melasma skin. A moderate-to-severe degree of solar elastosis was reported in 93% of melasma patients [8]. A significantly higher degree of solar elastosis was observed in lesional melasma skin compared with perilesional skin (83% *vs.* 29%, *p* < 0.05) [9]. The amount of elastotic material in lesional melasma skin was higher than that in perilesional skin (13.3% ± 2.8% *vs.* 10.2% ± 2.9%, *p* < 0.001) [10]. Histologically, thick, highly curled, and more fragmented elastic fibers were observed in Verhoeff–van-Gieson-stained sections of melasma skin [8]. In summary, 83% to 93% of melasma patients showed a variable degree of solar elastosis with an abnormal and irregular shape of elastotic material.

The higher level of solar elastosis in melasma skin, despite the variations, suggests that photoaging plays a crucial role in the development of melasma. Ultraviolet B (UVB) irradiation promote keratinocytes to induce melanocyte proliferation and melanogenesis by secreting stem cell factor (SCF), basic fibroblast growth factor (bFGF), interleukin-1, endothelin-1, inducible nitric oxide synthase, an α-melanocyte-stimulating hormone, an adrenocorticotropic hormone, and prostaglandin E2 [11,12,13,14,15]. Furthermore, solar damage of the skin may induce melanogenic cytokines, including SCF and hepatocyte growth factor, from the dermal fibroblasts, thereby influencing the development of hyperpigmentation in the overlying epidermis [16,17].

Transcriptional profiling revealed that a subset of Wnt signaling modulators, including Wnt inhibitory factor-1 (WIF-1), secreted frizzled-related protein 2 (sFRP2), and Wnt5a, were upregulated in lesional melasma skin [18]. The upregulation of WIF-1 on cultured normal human melanocytes significantly promoted melanogenesis by inducing expressions of microphthlamia-associated transcription factor (MITF) and tyrosinase [19]. WIF-1 downregulation, which may occur in epidermal keratinocytes and in dermal fibroblasts, is involved in melasma development through upregulation of the canonical and the noncanonical Wnt signaling pathway [20]. sFRP2 has been investigated to be overexpressed in melasma or UV-irradiated skin to stimulate melanogenesis through MITF or tyrosinase upregulation via β-catenin signaling [21]. Furthermore, pleiotrophin, a heparin-binding protein reflecting cell aging, was hypothesized to be associated with melanogenesis, likely through MITF degradation via Erk1/2 activation in melanocytes [22].

### 2.2. Basement Membrane Disruption

Abnormalities in the basement membrane of melasma skin have been described in several studies. For example, the presence of vacuolar degeneration of the basal cells and focal vacuolar degeneration of the basement membrane was reported in 3.9% (3/76) of melasma skin specimens [7]. Pendulous melanocytes associated with basement membrane abnormalities were demonstrated as a characteristic feature of melasma [23]. These findings suggest that the basement membrane disruption is an additional key finding for melasma. Interestingly, compared with the low incidence in the earlier study, a recent study of melasma patients with Fitzpatrick skin types IV and V revealed a disrupted basement membrane in 95.5% and 83% of skin samples via periodic acid-Schiff-diastase (D-PAS) staining and anti-collagen type IV immunohistochemistry, respectively [9]. D-PAS staining is a well-known histochemical staining for the basement membrane, and type IV collagen is the main component of the basement membrane. Although there may be a huge variation in the incidence of basement membrane disruption in the literature depending on the study population (3% to 95.5%), basement membrane disruption is an important finding, as it reveals the relationship between chronic UV exposure and melasma. During chronic UV exposure, elevated levels of matrix metalloproteinase (MMP)-2 and MMP-9, which degrade type IV collagen and type VI collagen in the skin, induce basement membrane disruption [24]. Further studies are necessary to confirm the prevalence of basement membrane disruption.

Basement membrane disruption facilitates the descent of melanocytes and melanin into the dermis, which would appear as free melanin or melanophages frequently observed in the dermis of melasma skin [8,9]. As a result, melasma is often refractory to treatment with high likelihood of recurrence [7]. Since basement membrane disruption is an additional cause of melasma recurrence, a restoration technology of the basement membrane will be necessary for long-term management of melasma.

### 2.3. Increased Vascularization

Accumulating evidence has demonstrated that the number of blood vessels, vessel size, and vessel density is greater in lesional melasma skin than in perilesional skin [25,26,27]. An immunohistochemical study of factor VIIIa-related antigen demonstrated an overall increase of 68.75% in the cutaneous area covered by blood vessels in melasma skin compared with peri-lesional normal skin [25].

Since the functioning vascular endothelial growth factor (VEGF) receptor was identified in melanocytes *in vitro*, the elevated VEGF in keratinocytes has been hypothesized to play a role in the elevated vascularization of melasma [28]. However, to date, there lacks evidence that VEGF is a strong melanogenic factor. Instead, increased vasculature is a consequence of solar elastosis induced by chronic UV exposure. Elevated level of c-kit—a well-known, strong melanogenic cytokine—is associated with solar elastosis, which consequently increases the melanogenesis of the overlying epidermis [29]. Furthermore, elevations in the levels of cytokines that could affect vascularization, such as SCF and inducible nitric oxide synthase, have also been demonstrated [29,30]. Since increased vascularization is regarded as a characteristic change of the aging process, melasma is considered as a unique phenotype of photodamaging during the aging process, rather than a pigmentary epidermal disorder. Thus, based on this reasoning, anti-aging and anti-angiogenic treatment should be considered for melasma.

### 2.4. Increased Number of Mast Cell

Mast cells are more frequently observed in melasma skin than in non-lesional skin, especially in the dermal elastotic areas [31]. The number of dermal mast cells was significantly higher in lesional melasma skin than in perilesional skin (173% ± 57% *vs.* 145% ± 57%, *p* = 0.04) [10]. By immunohistochemical staining, the number of mast cells detected was 58 ± 39.9 cells/mm^2^ in melasma skin, compared with 37 ± 28.8 cells/mm^2^ in perilesional skin (*p* < 0.04) [9]. However, large standard deviations of these data imply that there is a big variation in the number of mast cells depending on the sample sites.

Although the role of mast cells in the development of melasma is not clearly understood, based on a few previous studies, histamine has been shown to act in the melanogenesis. The release of histamine in the human dermal mast cells is upregulated in a response to UV irradiation [32,33]. Histamine stimulates the proliferation and migration of melanocytes [34]. The melanogenic activity of histamine is mediated by H2 receptors via protein kinase A activation [35]. The growth-differentiation factor-15, a member of transforming growth factor-β (TGF-β) family, has recently been suggested to play a role in the histamine-induced melanogenesis [36]. Thus, mast cells may initiate epidermal pigmentation, which is the main feature of melasma.

**Figure 1:**
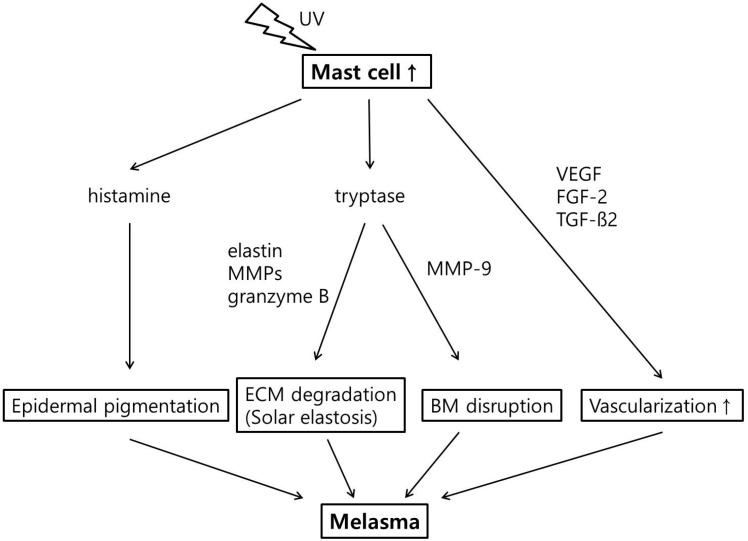
The role of mast cells in melanogenesis and photoaging. UV= ultraviolet; MMPs = matrix metalloproteases; VEGF = vascular endothelial growth factor; FGF-2 = fibroblast growth factor-2; TGF-β = transforming growth factor-β; ECM = extracellular matrix; BM = basement membrane.

Furthermore, the relationship between mast cells and photoaging has been investigated in several studies (Figure 1). It has been shown that the number of mast cells is significantly increased in photoaged skin [37,38]. Repetitive UV irradiation also promotes the production of mast cell tryptase, which has been demonstrated to be involved in dermal ECM degradation by processing proMMP to active forms or directly damaging ECM proteins [39,40,41,42]. Mast cell tryptase activates proMMP-9 and degrades type IV collagen [43,44]. Thus, elevated mast cell numbers and tryptase levels could weaken the basement membrane in melasma skin [44]. Mast cell tryptase could also trigger solar elastosis by inducing the production of elastin by fibroblasts, either directly or via other cell types or cytokines [45,46]. Interestingly, solar elastosis did not develop in mast cell-deficient mice that were repeatedly irradiated with UV [47]. A recent study further revealed that granyme B, a serine protease expressed by increased mast cell population, contributes to ECM degradation in the skin after UV irradiation [48]. Finally, mast cells can also induce vascular proliferation by secreting angiogenic factors, including VEGF, fibroblast growth factor-2 (FGF-2), and TGF-β [49]. These findings indicate that mast cells play a key role in chronic UV-induced photoaging and are associated with solar elastosis, basement membrane disruption, and vascular dilatation, all of which are predominant features of melasma.

## 3. Clinical Implications from the Histology of Melasma

### 3.1. Topical Treatments

Topical treatment is still the main mode of treatment for hyperpigmentary conditions, including melasma. Hydroquinone (HQ), the most popular anti-melanogenic agent, inhibits the conversion of l-3,4-dihydroxyphenylalanine to melanin by competitively inhibiting tyrosinase, the rate-limiting enzyme in the process of melanogenesis [50]. Oxidative products from HQ could also damage membrane lipids and proteins, including tyrosinase [51].

Unfortunately, safety issues surrounding HQ are still controversial. The European Committee prohibited the use of HQ in cosmetics due to its potential complications, known as exogenous ochronosis and permanent depigmentation [52]. Moreover, issues were raised for its potential carcinogenic risk from the metabolites, *p*-benzoquinones, which are formed in the liver [53]. In addition, rhododenol, a newly introduced tyrosinase inhibitor, has been reported to induce vitiligo, which is one of the intractable skin diseases [54]. These suggest that more safe and effective topical ingredients need to be developed.

Even though topical treatment can decrease melanogenesis, pigmentary conditions, as aforementioned, will frequently recur since the surrounding conditions that affect melanogenesis in melasma still remain. Then, topical treatment needs to be combined with an anti-aging approach, such as topical tretinoin or laser or light therapy. A triple combination cream (TCC) contains 4% HQ, 0.05% tretinoin, and 0.01% fluocinolone acetonide [50]. TCC is the only HQ-containing drug which was approved by the United States Food and Drug Administration (FDA) for the treatment of melasma [50,55,56]. Tretinoin displays not only an anti-wrinkle effect, but also a hypopigmenting property [57]. Steroids inhibit the secretion of endothelin-1 and granulocyte macrophage colony-stimulating factor, which are involved in UV-induced melanogenesis [58,59].

### 3.2. Systemic Treatments

Ascorbic acid, a well-known antioxidant compound, binds with copper of tyrosinase to inhibit tyrosinase activity and suppress oxidative polymerization of melanin intermediates [60,61,62]. Other oxidant compounds, such as α-tocopherol, hydrocoumarins, and thioctic acid, also have anti-melanogenic effects *in vitro* [63,64,65]. Although antioxidants have been described to be somewhat beneficial in the treatment for melasma, no standard oral regimen has been established thus far.

**Figure 2:**
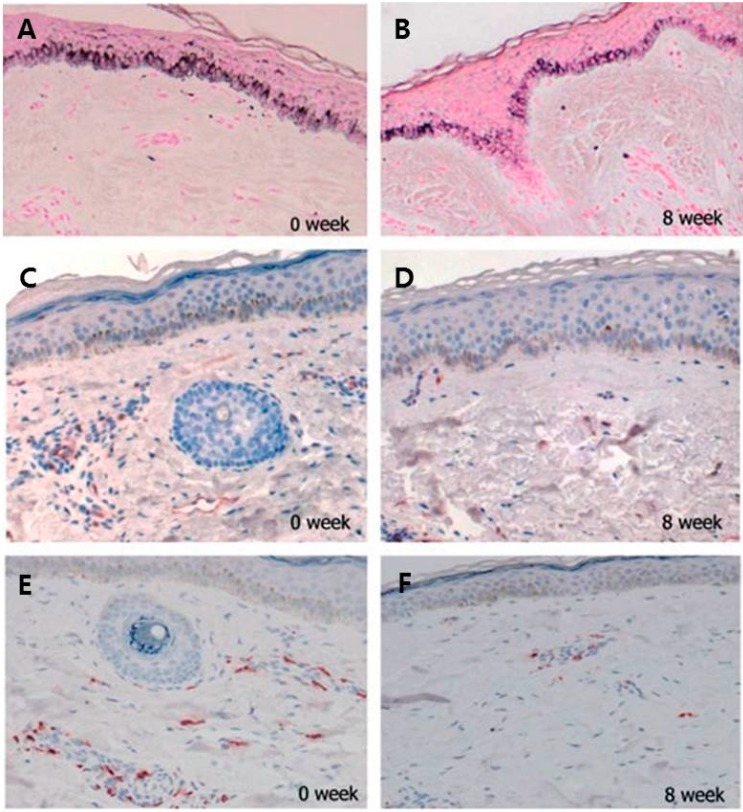
Histologic changes after eight weeks of treatment with tranexamic acid. (**A**,**B**) Fontana–Masson staining shows reduced epidermal pigmentation (×100); (**C**,**D**) Anti-CD31 staining shows reduced levels of vascularity (×100); and (**E**,**F**) Antitryptase staining shows reduced mast cell numbers (×100). Reproduced from [31].

Tranexamic acid (TXA) inhibits plasmin, which converts extracellular matrix-bound VEGF into its free forms [66]. TXA also suppresses neovascularization-induced bFGF [67]. Based on previous reports in the literature, TXA can be an effective treatment for melasma; however, there lacks a well-performed study that precisely shows the effects of TXA with a clear explanation of its mechanism. In a recent clinical trial that evaluated the efficacy of systemic TXA as the treatment for melasma, we demonstrated a significant decrease in the lesional melanin index and a decrease in the erythema index after an oral administration of 250 mg of TXA, three times per day for eight weeks [31]. A histologic analysis showed significant reductions not only in the level of epidermal pigmentation, but also in the number of mast cells and vessels (Figure 2). Our results suggest that systemic treatment of melasma may require an anti-aging approach through mast cells and accompanying dermal degenerative changes, including vascular dilatation.

### 3.3. Laser and Light Therapies

Numerous studies have demonstrated that laser or light therapy, including intense-pulsed light, the fractional 1550-nm non-ablative laser, the Q-switched neodymium-doped yttrium aluminum garnet laser (QSNYL), the pulsed-dye laser, and the copper bromide laser, has shown positive efficacy as treatment for melasma [68,69,70,71,72]. The “laser-toning” technique using a collimated, low-fluence, 1064-nm QSNYL and is one of the first-line therapies for melasma in East Asian countries. Its mechanism of action is unclear. However, several studies have demonstrated that the laser-toning technique removed melanosomes without damaging melanocytes [73]. Moreover, this technique damaged the dendrites of the melanocytes without destructing whole cells [74]. Therefore, the laser-toning technique inhibits the melanocytes activity by a process called “subcellular selective photothermolysis” [73,74].

Although laser-toning could decrease pigmentation without post-inflammatory hyperpigmentation, a high recurrence of pigmentation is anticipated since laser toning cannot remove the underlying dermal pathology. Recently, depigmented mottling lesions, in which dormant melanocytes were observed, have been reported from repeated laser-toning [75]. This suggests that the accumulation of high energy via repetitive laser-toning induces a scar-like condition that hampers melanogenesis by melanocytes.

There are some reports in the literature that assert that the vascular laser may not be effective as a treatment for melasma [71]. However, through repeated trials, it will remove abnormal vascular structures and surrounding degenerative changes.

### 3.4. Chemical Peels

Chemicals peels are usually applied in the treatment of melasma in Caucasians [76]. However, the result was unsatisfactory in Asian patients with Fitzpatrick skin types III–IV because of a high risk of adverse effects, especially post-inflammatory hyperpigmentation. Thus, chemical peels are not preferred in the treatment of Asian melasma patients. Only a few selected recalcitrant patients receive this mode of treatment.

## 4. Conclusions

Heterogeneous histologic findings of melasma provide clues to the pathogenesis of the disease. Dermal ECM abnormality, especially solar elastosis, revealed that photoaging plays a crucial role in the development of melasma via melanogenic cytokines and the Wnt signaling pathway. Basement membrane disruption caused by elevated levels of MMP-2 and MMP-9 facilitates the descent of melanocytes into dermis, which makes the treatment of melasma challenging. Increased vascularization in melasma is a consequence of chronic UV exposure, which requires anti-aging and anti-angiogenic treatment. Finally, an increased number of mast cells plays a key role in the development of melasma and is associated with solar elastosis, basement membrane disruption, and vascular dilatation. Since melasma is often recalcitrant to treatment and often recurs despite successful treatment, the treatment of melasma is difficult. Though topical HQ is still the treatment of choice in melasma, anti-aging approaches, such as TXA, to correct dermal degenerative changes and an increased number of mast cells has been proposed.

## Abbreviations

The following abbreviations are used in this manuscript:

UV	ultraviolet
ECM	extracellular matrix
SCF	stem cell factor
bFGF	basic fibroblast growth factor
WIF-1	Wnt inhibitory factor-1
sFRP2	secreted frizzled-related protein 2
MITF	microphthlamia-associated transcription factor
D-PAS	periodic acid-Schiff-diastase
MMP	matrix metalloproteinase
VEGF	vascular endothelial growth factor
TGF-β	transforming growth factor-β
FGF-2	fibroblast growth factor-2
HQ	hydroquinone
TCC	triple combination cream
TXA	tranexamic acid
QSNYL	Q-switched neodymium-doped yttrium aluminum garnet laser
